# Protein C activity as a potential prognostic factor for nursing home-acquired pneumonia

**DOI:** 10.1371/journal.pone.0274685

**Published:** 2022-10-12

**Authors:** Issei Oi, Isao Ito, Naoya Tanabe, Satoshi Konishi, Nobuyoshi Hamao, Masahiro Shirata, Seiichiro Imai, Yoshiro Yasutomo, Seizo Kadowaki, Hisako Matsumoto, Yu Hidaka, Satoshi Morita, Toyohiro Hirai

**Affiliations:** 1 Department of Respiratory Medicine, Graduate School of Medicine, Kyoto University, Kyoto, Kyoto, Japan; 2 Department of Internal Medicine, Ono Municipal Hospital, Ono, Hyogo, Japan; 3 Department of Biomedical Statistics and Bioinformatics, Graduate School of Medicine, Kyoto University; Istanbul University-Cerrahpasa, Cerrahpasa Medical Faculty, TURKEY

## Abstract

**Introduction:**

Despite the poor prognosis for nursing home acquired pneumonia (NHAP), a useful prognostic factor is lacking. We evaluated protein C (PC) activity as a predictor of in-hospital death in patients with NHAP and community-acquired pneumonia (CAP).

**Methods:**

This prospective, observational study included all patients hospitalized with pneumonia between July 2007 and December 2012 in a single hospital. We measured PC activity at admission and investigated whether it was different between survivors and non-survivors. We also examined whether PC activity < 55% was a predictor for in-hospital death of pneumonia by logistic regression analysis with CURB-65 items (confusion, blood urea >20 mg/dL, respiratory rate >30/min, and blood pressure <90/60 mmHg, age >65). When it was a useful prognostic factor for pneumonia, we combined PC activity with the existing prognostic scores, the pneumonia severity index (PSI) and CURB-65, and analyzed its additional effect by comparing the areas under the receiver operating characteristic curves (AUCs) of the modified and original scores.

**Results:**

Participants comprised 75 NHAP and 315 CAP patients. PC activity was lower among non-survivors than among survivors in NHAP and all-pneumonia (CAP+NHAP). PC activity <55% was a useful prognostic predictor for NHAP (Odds ratio 7.39 (95% CI; 1.59–34.38), and when PSI or CURB-65 was combined with PC activity, the AUC improved (from 0.712 to 0.820 for PSI, and 0.657 to 0.734 for CURB-65).

**Conclusions:**

PC activity was useful for predicting in-hospital death of pneumonia, especially in NHAP, and became more useful when combined with the PSI or CURB-65.

## Introduction

The world is aging and aging of the population has led to an increase in the number of disabled people [1]. Many of them have to enter a nursing home [2], and pneumonia is one of the most common infections identified in nursing home residents [3]. Pneumonia occurring in nursing homes (Nursing home-acquired pneumonia, NHAP) has been considered to be distinct from community-acquired pneumonia (CAP) with unique epidemiological features and poor outcomes [4–11] since its proposal in 1978 [12]. The mortality of NHAP ranges from 6.5 to 40% [4,6], and it is as high as 18.1% in Japan [13].

Various severity scoring systems have been developed for CAP patients and several useful biomarkers have been proposed [14–16]. In NHAP, Naughton et al. [17] developed the severity scoring system for NHAP, but Lee et al. [18] confirmed that it was less useful than the pneumonia severity index (PSI) [14] and CURB-65 score (confusion, blood urea >20 mg/dL, respiratory rate >30/min, blood pressure <90/60 mmHg, age >65) [15]. Procalcitonin has potential as the prognostic predictor for NHAP [19], but one study revealed that procalcitonin may serve as a poor marker in NHAP [20], and its evaluation is yet to be determined.

The relationship between severe infection and coagulation-fibrinolytic systems has long been of interest [21], and some coagulation-fibrinolytic markers have been found to correlate clinically with the prognosis of severe sepsis [22]. Protein C (PC) is a protein comprising 461 amino acids with a molecular weight of 55 kDa, activated by thrombomodulin [23]. PC is known to act on the immune system as well as to be a modulator of the coagulation system [24]. Previous studies have reported that decreased PC activity was associated with the prognosis of severe sepsis [25–32] and acute respiratory distress syndrome (ARDS)/acute lung injury (ALI) [33–35], but the relationship between PC activity and the prognosis of pneumonia remains unknown. The present study aimed to measure PC activity at hospitalization for pneumonia and evaluate whether PC activity can be a useful prognostic factor for CAP and NHAP.

## Materials and methods

### Design and patients

This study was a prospective, observational study that included all patients who were hospitalized with pneumonia between July 2007 and December 2012 at Ono Municipal Hospital, Hyogo, Japan.

This study enrolled hospital-admitted patients ≥15 years old with a diagnosis of pneumonia requiring initial parenteral treatment. Pneumonia was diagnosed by the radiological appearance of a new and/or progressive pulmonary infiltrate and greater than or equal to any two of the following conditions: documented axillary body temperature ≥37.5°C within the past 24 h; rigors and/or chills; general malaise, cough, sputum or change of sputum character (increased volume and/or purulence); tachypnea, dyspnea, rales consistent with the lung infiltrate; and WBC count ≥10,000/mm^3^ or <3,000/mm^3^ [36].

CAP was defined as a diagnosis of pneumonia among patients living in the community, while patients residing in a nursing home or long-term care facility with pneumonia were categorized as NHAP. The term ‘nursing home’ included special elderly nursing homes, geriatric nursing and healthcare facilities, medical facilities for nursing and recuperation, as well as geriatric nursing and healthcare facilities for recuperation. We excluded patients with any of the following: hospital-acquired pneumonia; pregnancy or breast-feeding; immunocompromising disease or receipt of immunocompromising therapy; active lung cancer; terminal illness; or other infiltrative diseases such as radiation pneumonitis, organizing pneumonia, drug-induced pneumonia, and obstructive pneumonia, tuberculosis, fungal infection, or empyema.

Baseline assessments included information on age, sex, residence, PSI score, CURB-65 score, and comorbid illness. Venous and arterial blood samples were obtained from each patient on admission to the hospital. Venous blood was analyzed for complete blood count and biochemistry; arterial blood was used for blood gas analysis. About 1.8 mL of blood was added to 0.2 mL of 3.2% sodium citrate, mixed by inverting 5 to 6 times, and then the plasma was separated immediately. Serum PC activity was measured in peripheral blood using commercial kits (Roche Diagnostics K.K., Tokyo, Japan). The assay range of PC activity was 10–150% and the reference standard range was 70–130%. PSI and CURB-65 scores were calculated as the existing severity of pneumonia using collected data, as previously reported [14,15]. All patients were followed until they were discharged alive or suffered an in-hospital death. We evaluated whether PC activity was useful for predicting an in-hospital death for patients with CAP or NHAP.

### Ethics

This study was approved by the institutional review board of Ono Municipal Hospital (19–6). Written informed-consent was obtained from all patients or their parents or guardians in cases of minors or patients who had difficulty consenting because of dementia, neurological disorders, or disturbance or consciousness.

### Statistical analysis

The primary response variable was a binary endpoint, that was survival discharge or an in-hospital death. The duration of hospitalization in elderly patients with pneumonia tends to be long, which leads to debilitation. We considered that debilitation and its resulting death were an important aspect of pneumonia in the elderly. Therefore, we set death or survival as the primary response variable, regardless of their hospitalization duration. Patients in our suburban municipal hospital were followed until they were discharged alive or suffered an in-hospital death.

During the study period, hospitalized cases were prospectively accumulated, bringing the total number of cases for analysis to 465. According to Peduzzi et al [37], the development of a logistic regression model requires the collection of at least 10 events per potential predictor; the proportion of in-hospital deaths due to pneumonia, including CAP and NHAP, was estimated to be 15%. Thus, at least 60 events were needed to develop a logistic regression model consisting of six potential predictors. This resulted in a required sample size of at least 400 patients (60×100/15). From these calculations, it was decided that the number of collected cases was sufficient for the analysis.

For the baseline clinical characteristics of enrolled patients, continuous variables were indicated as the mean ± standard deviation. The Mann-Whitney U test was used to compare the medians of continuous variables (such as age) and the chi-square test or Fisher’s exact test was used to compare the proportions of categorical variables (such as sex). The significance level was defined as p <0.05 for two-sided tests.

In this study, we first evaluated the usefulness of the PSI and CURB-65 for predicting in-hospital deaths. For each CURB-65 and PSI score, the number and percentage of in-hospital deaths in patients with overall pneumonia, CAP, and NHAP were calculated. We then calculated the areas under the curve (AUC) using the receiver operating characteristic (ROC) curves and examined the predictive ability of each score for the outcome of death.

Second, to examine whether PC is a useful predictor of in-hospital death, we compared differences between survivors and non-survivors using the Mann-Whitney U test. We then examined the ability of continuous CURB-65 items (age, systolic blood pressure, respiratory rate, and blood urea nitrogen) and PC activity to predict an outcome of death. Multiple logistic regression analysis was performed with the outcome of death, CURB-65 items and PC activity as predictors. Although both the PSI and CURB-65 are widely and clinically used as prognostic scoring instruments, we selected the clinical laboratory values of CURB-65 as a covariate because the PSI is complicated by the need for arterial blood gas analysis and overlaps with CURB-65 on many items. PC activity was categorized by cutoff values from the Youden index of the ROC curve.

Finally, we evaluated whether the addition of PC activity to the PSI and CURB-65 could predict the prognosis of NHAP by calculating the AUC. Since the PSI and CURB-65 are already well-known severity scoring of pneumonia, we did not change the cutoff values or add points for future clinical application. Because the PSI is a composite scoring of various factors, we tested the addition of a score for positive PC activity at the optimal cutoff value. All statistical analyses were performed using JMP version 14.0.0 software (SAS Institute Inc., Cary, NC).

## Results

### Patient characteristics

During the study period, 465 patients with pneumonia were hospitalized. Data on PC activity were missing for 74 of these patients because we could not take measurements due to hospitalization on holidays, and other laboratory data were missing in one patient. Subsequently, 315 CAP cases and 75 NHAP cases were analyzed for this study.

Baseline characteristics of cases with CAP and NHAP are presented in Table 1. Patients with NHAP were older (p<0.0001) and had higher PSI scores (p<0.0001). Blood urea nitrogen (BUN) was significantly higher and albumin levels were significantly lower in NHAP cases than in CAP cases (p = 0.022 and p<0.0001, respectively). There was no significant difference in PC activity between CAP and NHAP cases (p = 0.081).

**Table 1:** Baseline clinical characteristics of enrolled patients.

		CAP (n = 315)	NHAP (n = 75)	p-value
Age (years)		74.0±17.4	87.1±6.7	<0.0001
Male (%)		185 (58.7)	33 (44.0)	0.021
PSI score		90.3±34.0	126.8±26.8	<0.0001
Class	I (%)	32 (10.2)	0	<0.0001
	II (%)	52 (16.5)	0	
	III (%)	83 (26.3)	2 (2.7)	
	IV (%)	111 (35.2)	43 (57.3)	
	V (%)	37 (11.7)	30 (40.0)	
CURB-65	0	52 (16.5)	1 (1.3)	0.002
	1	121 (38.4)	25 (33.3)	
	2	98 (31.1)	29 (38.7)	
	3	31 (9.8)	16 (21.3)	
	4	12 (3.8)	4 (5.3)	
	5	1 (0.3)	0	
BT (°C)		38.1±0.9	38.0±1.0	0.096
HR (beats/min)		88.3±17.0	92.8±20.0	0.119
RR (breaths/min)		20.8±5.7	23.2±7.4	0.021
sBP (mmHg)		132.1±24.4	132.3±27.1	0.651
WBC (×10^3^/μL)		11.4±5.1	12.3±6.7	0.827
CRP (mg/dL)		10.2±7.5	8.8±7.4	0.095
PC activity (%)		70.3±23.5	67.2±21.8	0.081
BUN (mg/dL)		20.1±11.7	23.3±13.9	0.022
Cre (mg/dL)		0.94±0.5	0.85±0.4	0.134
Alb (mg/dL)		3.6±0.5	3.2±0.4	<0.0001
Ht (%)		36.2±5.5	35.2±4.5	0.037
Na (mEq/L)		137.3±8.1	136.8±7.4	0.344
Glu (mg/dL)		134.9±46.0	138.4±51.7	0.716
Primary antibiotics duration (days)		9.4±3.5	10.5±3.6	0.004
Deaths (%)		23 (7.3)	12 (16.0)	0.018

Values are presented as n (%) or mean ± standard deviation. CAP = community-acquired pneumonia; NHAP = nursing home-acquired pneumonia; PSI = pneumonia severity index; BT = body temperature; HR = heart rate; RR = respiratory rate; sBP = systolic blood pressure; WBC = white blood cells; CRP = C-reactive protein; PC = protein C; BUN = blood urea nitrogen; Cre = creatinine; Alb = albumin; Ht = hematocrit; Na = sodium; Glu = glucose.

The duration of primary antibiotic treatment was significantly longer in NHAP cases (10.5±3.6 days) than in CAP cases (9.4±3.5 days, p = 0.004). Mortality was 7.3% in CAP cases and 16.0% in NHAP cases (p = 0.018).

### Performance of severity scoring systems in pneumonias

**Fig 1:**
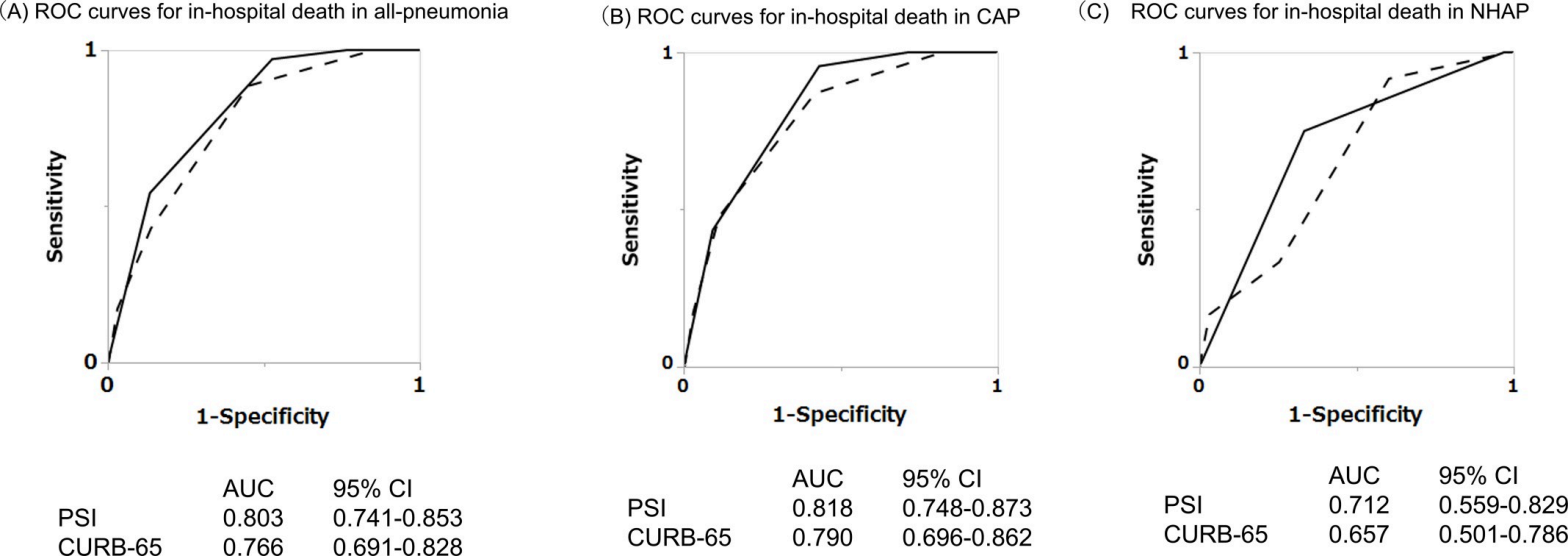
ROC curves of PSI or CURB-65 for predicting in-hospital death in all-pneumonia, CAP, and NHAP cases. Solid lines represent the ROC curves of PSI classes. Dashed lines represent the ROC curves of CURB-65 scores. The AUCs are shown at the bottom of each figure. The AUCs of both PSI and CURB-65 are sufficient to predict the in-hospital deaths in all-pneumonia.

The number of cases and mortalities across severity classes of PSI and CURB-65 are shown in Table 2. Both PSI classes and CURB-65 scores were significantly higher among patients who died than among survivors of CAP (p<0.001, for both), while only PSI classes had a significant difference among survivors and non-survivors of NHAP (p<0.001). The ROC curves of the PSI and CURB-65 for in-hospital deaths in CAP or NHAP cases are shown in Fig 1. AUC for both PSI and CURB-65 were sufficient for CAP (0.818 for PSI, and 0.790 for CURB-65). For all-pneumonia, the AUCs were also sufficient: 0.803 for PSI and 0.766 for CURB-65.

**Table 2:** The prognostic power of PSI and CURB-65 for in-hospital death of pneumonia.

I) PSI						
Number	Class	1	2	3	4	5
	All	32	52	85	154	67
	CAP	32	52	83	111	37
	NHAP	0	0	2	43	30
Deaths (%)	All	0	0	1 (1.2)	15 (9.7)	19 (28.4)
	CAP	0	0	1 (1.2)	12 (10.8)	10 (27.0)
	NHAP	0	0	0	3 (7.0)	9 (30.0)

II) CURB-65							
	Score	0	1	2	3	4	5
Number	All	53	146	127	47	16	1
	CAP	52	121	98	31	12	1
	NHAP	1	25	29	16	4	0
Deaths (%)	All	0	4 (2.7)	16 (12.6)	9 (19.2)	6 (37.5)	0
	CAP	0	3 (2.5)	9 (9.2)	7 (22.6)	4 (33.3)	0
	NHAP	0	1 (4.0)	7 (24.1)	2 (12.5)	2 (50.0)	0

CAP = community-acquired pneumonia; NHAP = nursing home-acquired pneumonia; PSI = pneumonia severity index.

### The differences between survivors and non-survivors in NHAP and all-pneumonia

The differences between survivors and non-survivors of NHAP and all-pneumonia are shown in Table 3. Non-survivors with NHAP had lower PC activity (p = 0.014) and higher blood urea nitrogen (BUN) levels (p = 0.004). There was no significant difference in age or albumin levels between survivors and non-survivors for NHAP (p = 0.557 and 0.794, respectively).

**Table 3:** Comparison between survivors and non-survivors in NHAP and all-pneumonia.

	NHAP			all-pneumonia		
	Survivors (n = 63)	Non-survivors (n = 12)	p-value	Survivors (n = 355)	Non-survivors (n = 35)	p-value
Age (years)	87.0±6.5	87.7±7.8	0.557	75.6±17.2	86.1±6.2	0.0001
Male (%)	28 (44.4)	5 (41.7)	0.859	196 (55.2)	22 (62.9)	0.385
PSI score	122.5±22.8	149.3±35.1	0.006	93.1±33.1	139.7±35.0	<0.0001
PSI Class	4.3±0.5	4.8±0.5	0.007	3.3±1.2	4.5±0.6	<0.0001
CURB-65	1.9±0.9	2.4±0.9	0.069	1.5±1.0	2.5±0.9	<0.0001
BT (°C)	38.0±1.0	37.6±0.5	0.228	38.0±0.9	37.8±0.8	0.024
HR (beats/min)	92.6±20.8	94.0±15.7	0.665	88.7±17.6	94.2±18.2	0.073
RR (breaths/min)	23.0±6.6	24.3±11.0	0.795	20.9±5.6	25.3±9.2	0.013
sBP (mmHg)	132.1±26.3	133.3±32.6	0.977	131.8±24.5	136.0±28.7	0.354
WBC (×10^3^/μL)	12.1±6.7	13.4±7.0	0.418	11.5±5.5	12.5±5.2	0.194
CRP (mg/dL)	9.1±7.7	7.3±5.4	0.659	10.0±7.7	9.0±5.4	0.826
PC activity (%)	69.5±23.6	55.1±11.1	0.014	70.8±23.4	54.8±18.3	0.003
BUN (mg/dL)	21.9±13.9	30.8±11.5	0.004	19.7±11.6	31.4±13.1	<0.0001
Cre (mg/dL)	0.8±0.3	1.1±0.6	0.275	0.9±0.4	1.3±0.8	0.008
Alb (mg/dL)	3.2±0.4	3.1±0.7	0.794	3.6±0.5	3.2±0.5	0.0001
Ht (%)	35.3±4.6	34.7±4.6	0.623	36.1±5.4	35.4±4.9	0.312
Na (mEq/L)	136.2±6.2	140.0±11.7	0.233	137.1±7.9	138.7±8.9	0.599
Glu (mg/dL)	136.8±49.4	147.0±64.2	0.908	133.7±45.7	154.5±57.0	0.020

Values are presented as n (%) or mean ± standard deviation. CAP = community-acquired pneumonia; NHAP = nursing home-acquired pneumonia; PSI = pneumonia severity index; BT = body temperature; HR = heart rate; RR = respiratory rate; sBP = systolic blood pressure; WBC = white blood cells; CRP = C-reactive protein; PC = protein C; BUN = blood urea nitrogen; Cre = creatinine; Alb = albumin; Ht = hematocrit; Na = sodium; Glu = glucose.

### PC activity as a prognostic factor in pneumonia

**Fig 2:**
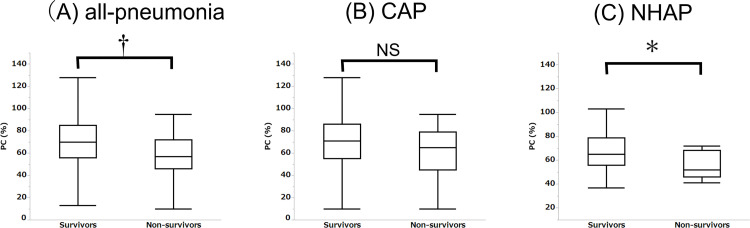
The difference in PC activity between survivors and non-survivors with pneumonias. Results are expressed as mean ± standard deviation. *p<0.05, ^†^p<0.01, comparing survivors and non-survivors. A significant difference was seen for NHAP cases (p = 0.014) but not for CAP (p = 0.077). In all-pneumonia, which combines CAP and NHAP, non-survivors showed significantly lower PC activity than survivors (p = 0.003).

PC activities of survivors and non-survivors of all-pneumonia, CAP, and NHAP are shown in Fig 2. PC activity was significantly lower in non-survivors of all-pneumonia (p = 0.003; Fig 2A). When CAP or NHAP were analyzed separately, PC activity was significantly lower in non-survivors of NHAP (p = 0.014; Fig 2C) but not of CAP (p = 0.077; Fig 2B).

### Evaluation of PC activity and each continuous variable of CURB-65 as prognostic factors

**Fig 3:**
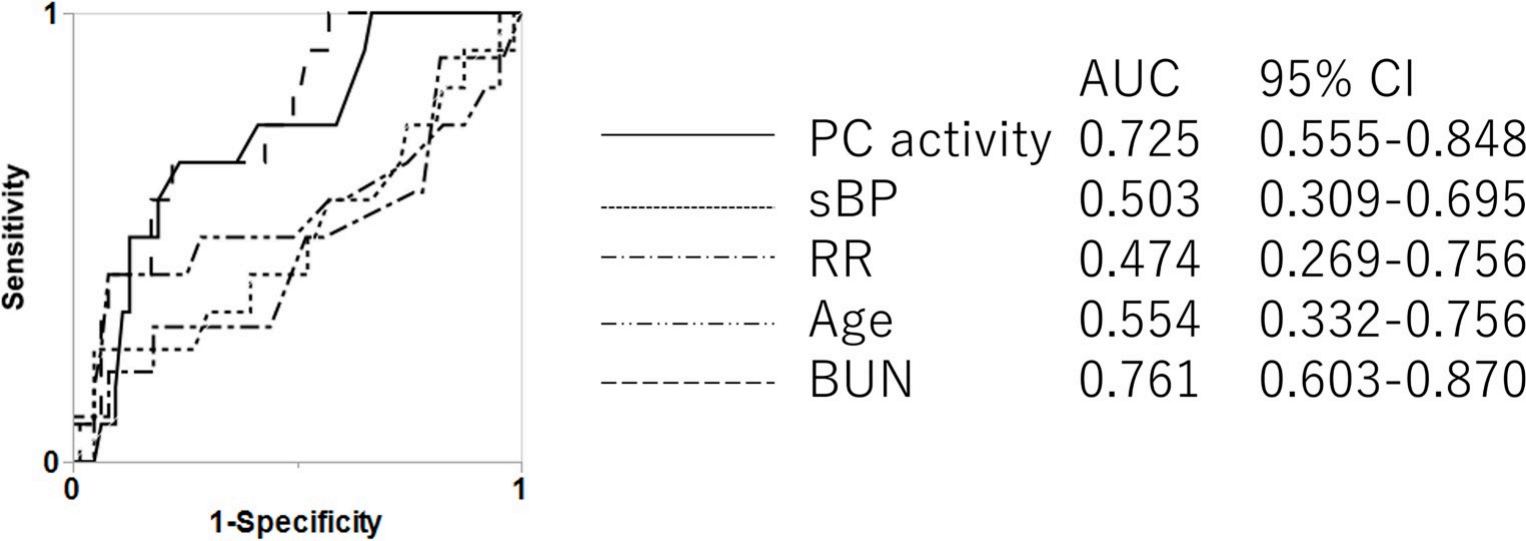
Evaluation of PC activity and each continuous variable of CURB-65 as predictors for in-hospital death of NHAP. PC = protein C; sBP = systolic blood pressure; RR = respiratory rate; BUN = blood urea nitrogen. *p<0.05, comparing AUCs. The ROC curves of PC activity or each item of CURB-65 in NHAP are shown. The AUC of PC activity is second highest after BUN. Significant differences are apparent between the AUC of BUN and that of systolic blood pressure (p = 0.035). There was no significant difference between the AUC of PC activity and those of the CURB-65 items.

**Fig 4:**
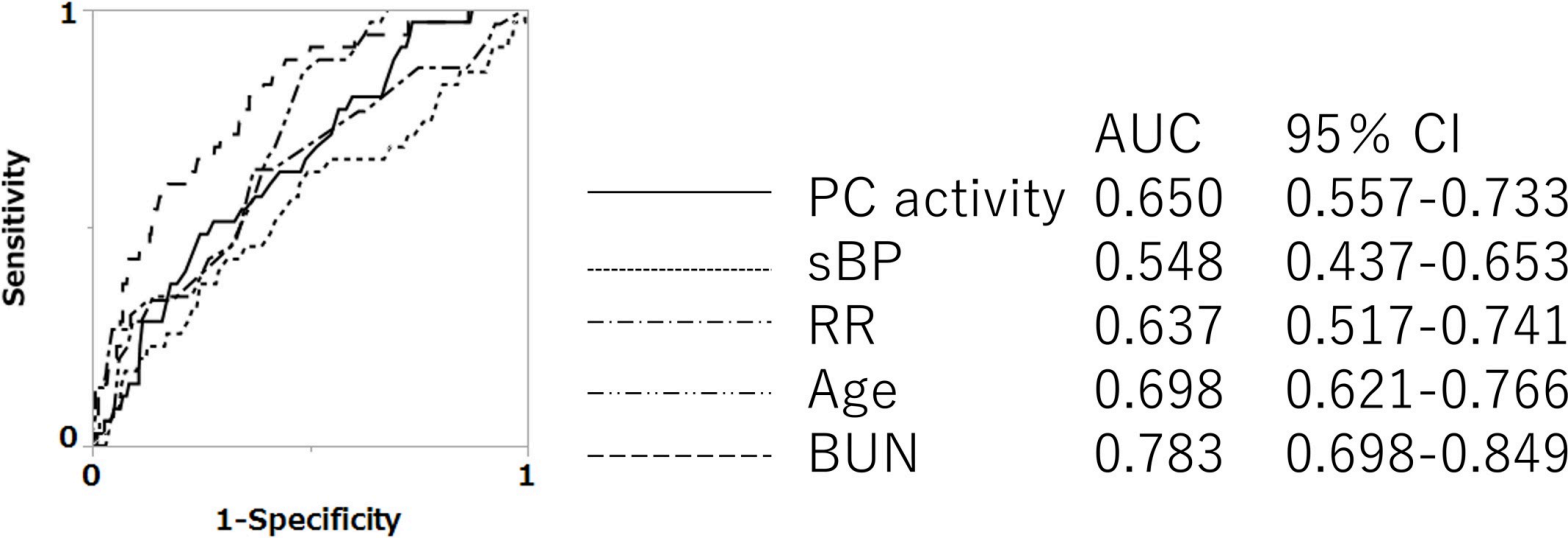
ROC curves of PC activity and each continuous variable of CURB-65 for predicting in-hospital death of all-pneumonia. PC = protein C; sBP = systolic blood pressure; RR = respiratory rate; BUN = blood urea nitrogen. *p<0.05, ^†^p<0.01, comparing AUCs. The ROC curves of PC activity or each item of CURB-65 in all-pneumonia are shown. In all-pneumonia, the AUC of PC activity was the third highest; BUN had the highest AUC. There were significant differences between BUN and systolic blood pressure, respiratory rate, and PC activity (p<0.01, <0.05, and <0.05, respectively).

The ROC curves of PC activity and each continuous variable of CURB-65 for mortality in NHAP cases are shown in Fig 3. The AUC was highest for BUN, and the AUC for PC activity was second highest for predicting mortality. For all-pneumonia, the AUCs for mortality of PC activity was 0.650 and this was the third highest following BUN and age. BUN showed the highest AUC for mortality of 0.783 (Fig 4).

### Optimal cut-off for PC activity

The optimal cut-off for PC activity to predict in-hospital mortality in NHAP, which was determined using Youden’s index of the ROC curve, was 54%; this offers 66.7% sensitivity and 76.2% specificity. For all-pneumonia, 55% was the optimal cut-off, providing 48.6% sensitivity and 75.2% specificity. Since optimal cut-offs for NHAP or all-pneumonia for mortality were around 55%, and the p-value for comparisons of PC activity between survivors and non-survivors were minimal for patients with all-pneumonia (p = 0.003, Fig 2), we adopted 55% as the optimal cut-off for further evaluation of PC activity with NHAP.

### PC activity as a prognostic factor for NHAP

Results of logistic regression analysis are shown in Table 4. The only useful predictor for in-hospital death in NHAP was PC activity <55% in multivariate analysis (Odds ratio (OR): 7.38, 95% confidence interval (CI): 1.59–34.36). When cut-off at 20 mg/dL as the item of CURB-65, BUN was not a prognostic predictor in NHAP.

**Table 4:** Examination of prognostic factors using logistic analysis in NHAP.

	Univariate	analysis		Multivariate analysis		
	survivor (n = 63)	non-survivor (n = 12)	p-value	OR	p-value	95% CI
Confusion	9 (14.3)	4 (33.3)	0.110	3.916	0.110	0.735–20.850
BUN >20 mg/dL	26 (41.3)	8 (66.7)	0.105	1.528	0.571	0.353–6.616
RR ≥30 breaths/min	9 (14.3)	2 (16.7)	0.831	1.259	0.817	0.179–8.840
sBP <90 mmHg or dBP ≤60 mmHg	12 (19.0)	3 (25.0)	0.637	0.615	0.598	0.101–3.745
Age ≥65 years	62 (98.4)	12 (100)	1.000	1.472×10^6^	0.994	0-inf.
PC activity <55%	15 (23.8)	8 (66.7)	0.003	7.383	0.011	1.586–34.359

NHAP = nursing home-acquired pneumonia; OR = odds ratio; CI = confidence interval; BUN = blood urea nitrogen; RR = respiratory rate; sBP = systolic blood pressure; dBP = diastolic blood pressure; PC = protein C. Each odds ratio in the multivariate analysis represents the probability that the variables are representative of non-survivors.

For all-pneumonia, BUN >20 mg/dL and respiratory rate ≥30 breaths/min were useful predictors of in-hospital death (OR: 4.34, 95%CI: 1.87–10.08 and OR: 2.93, 95%CI: 1.12–7.65, respectively), whereas PC activity <55% was not predictive in multivariate analysis (OR: 2.03, 95%CI: 0.95–4.34, Table 5).

**Table 5:** Examination of prognostic factors using logistic analysis in all-pneumonia.

	Univariate analysis			Multivariate analysis		
	survivor (n = 355)	non-survivor (n = 35)	p-value	OR	p-value	95% CI
Confusion	35 (9.9)	9 (25.7)	0.005	1.71	0.265	0.666–4.380
BUN >20 mg/dL	121 (34.1)	27 (77.1)	< .0001	4.34	0.0006	1.869–10.082
RR ≥30 breaths/min	25 (7.0)	8 (22.9)	0.001	2.93	0.028	1.121–7.650
sBP <90 mmHg or dBP ≤60 mmHg	56 (15.8)	8 (22.9)	0.280	1.11	0.830	0.431–2.851
Age ≥65 years	286 (80.6)	35 (100)	0.002	1.10×10^7^	0.993	0-inf.
PC activity <55%	84 (23.7)	16 (45.7)	0.004	2.03	0.067	0.953–4.342

OR = odds ratio; CI = confidence interval; BUN = blood urea nitrogen; RR = respiratory rate; sBP = systolic blood pressure; dBP = diastolic blood pressure; PC = protein C, inf. = infinity. Each odds ratio in the multivariate analysis represents the probability that the variables are representative of non-survivors.

### Additional effects of using PC activity with PSI or CURB-65 for prognosis of NHAP

In multivariate analysis, PC activity was a useful prognostic factor for NHAP, so we incorporated this into conventional prognostic methods. When PC activity was ≤54%, we added 20, 25 or 30 points to the original PSI score and re-examined the results. We identified the addition of 25 points as being the most appropriate for predicting in-hospital death in NHAP (AUCs: 0.7716, 0.7727, and 0.7725, respectively, for the addition of 20, 25, and 30 points in S1 Fig). To develop more effective predictors of NHAP, we reclassified the PSI scores with the addition of each point as the modified PSI class. Modified classes one to four were the same as the original PSI classes. In patients with a modified PSI score ≥130, the new class five was defined as 130–159 and the new class six as ≥160.

**Fig 5:**
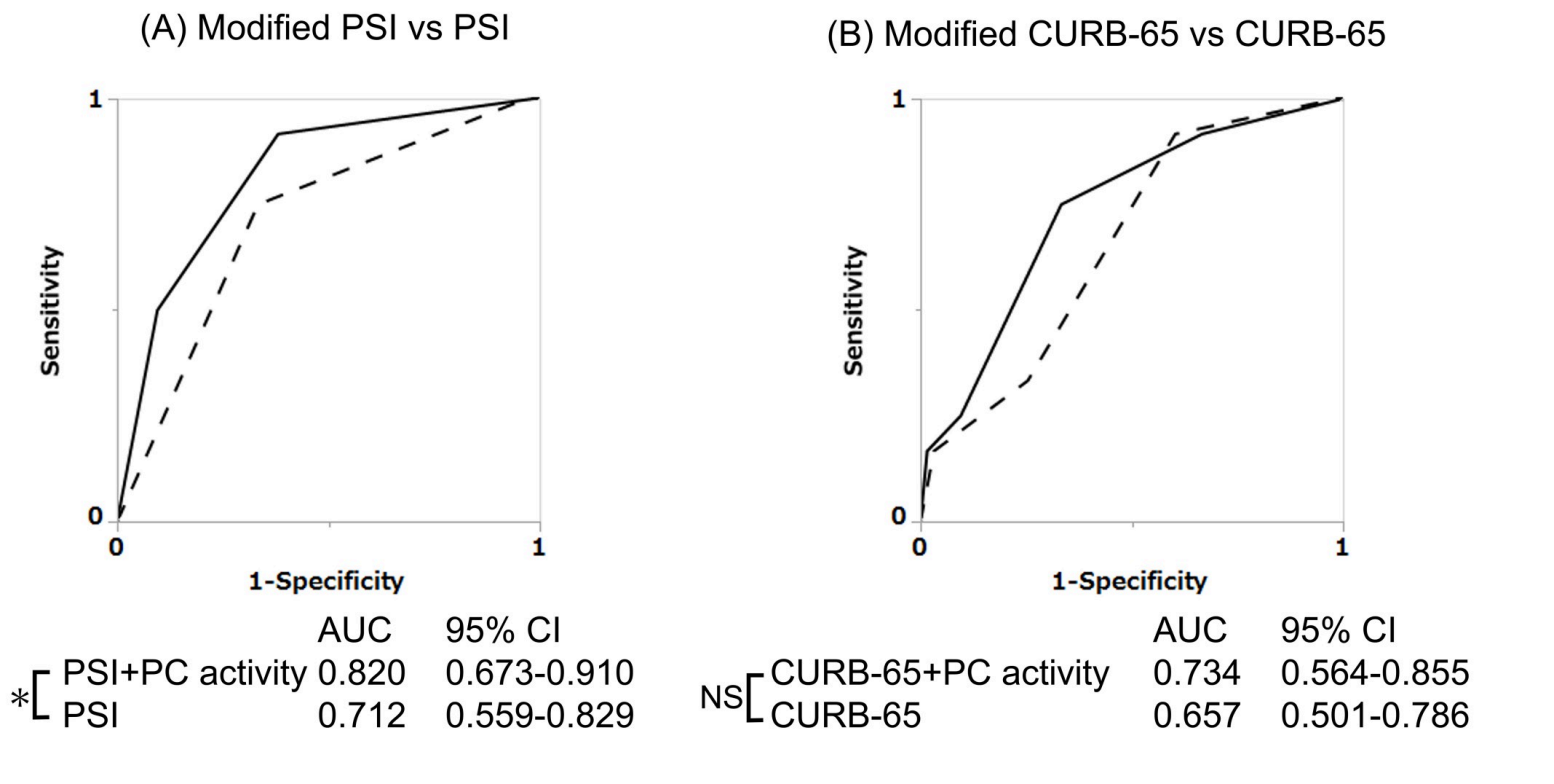
Additional effects of PC activity with PSI or CURB-65 for prognosis of NHAP. Dashed lines represent original PSI or CURB-65 as shown with solid lines in Fig 1. Solid lines are additional examination of PC activity. We added 25 points to the PSI score if the patient showed PC activity <55%, and reclassified the patient based on the rescored PSI into classes 1–6. Classes 1–4 are the same as in the original PSI classification. Patients with modified PSI scores ≥130 were divided into class 5 for scores within 130–159 or class 6 for scores ≥160. The modified PSI class had a significantly higher AUC for mortality compared to that of the original PSI classes (p = 0.036) (A). For CURB-65, 1 point was added to the CURB-65 when PC activity was <55%, making 6 the maximum total score (B).

The ROC curves of the original and modified PSI classes for mortality in NHAP are shown in Fig 5A. The AUC for mortality then improved from 0.712 for the original PSI to 0.820 for the modified PSI (p = 0.036). Fig 5B shows comparisons between the ROC curve for the original CURB-65 and that for the modified CURB-65 made by adding one point to the original CURB-65 for PC activity <55%, giving a maximum total score of six; the AUC increased from 0.657 to 0.734 (p = 0.062). The AUCs for PSI and CURB-65 thus improved across the board when combined with PC activity in NHAP.

## Discussion

In this study, the mortality of NHAP was higher than that of CAP. Existing scorings were found to be sufficient for CAP, so we explored prognostic factors that would be particularly useful especially for NHAP. Non-survivors showed lower PC activity than survivors in NHAP, but this was not the case in CAP. PC activity was suggested to be as useful as each continuous variable of CURB-65, and a cut-off at 55% allowed PC activity to be used as a valid predictor of prognosis for NHAP. Moreover, PSI and CURB-65 appeared effective for predicting prognosis in NHAP when combined with PC activity.

PC is a vitamin K-dependent protein discovered from bovine plasma in 1972 [38] and has been found to have various effects. PC is converted to activated PC (APC) by thrombin bound to thrombomodulin on endothelial cells. In conjunction with the cofactor protein S, APC acts as a coenzyme to inhibit factor Va and factor VIIIa, resulting in inhibition of factor X, activation by factor IXa, and activation of prothrombin by factor Xa [39,40]. In this manner, the production of thrombin is inhibited, and APC limits coagulation and regulates endogenous fibrinolytic activity. In addition, APC decreases thrombin-mediated proinflammatory cytokines [23], and as a result, reduces inflammatory reactions to infection. Moreover, a recent study indicated that APC directly modulates endothelial and leukocyte functions by triggering cell signaling mediated by sphingosine kinase-1 activity and activation of S1P1 signaling [41]. Decreased PC has been correlated with fatal diseases such as severe sepsis [25–32], ARDS/ALI [33–35], multiple-organ dysfunction syndrome [42] and trauma [43]. Based on the above, we hypothesized that PC activity on admission may be a useful prognostic marker in pneumonia.

NHAP patients are known to show a higher mortality rate than CAP patients [4–11], but useful prognostic predictors for NHAP are lacking. Moreover, the prognostic predictors for CAP such as albumin or C-reactive protein [44,45] were not predictors for NHAP (Table 3). The reason for this is that NHAP includes patients with various backgrounds, such as elderly individuals with poor activities of daily living and various comorbidities, and the factors determining the prognosis of NHAP are presumably more diverse than those of CAP. For such a wide variety of patients, identification of effective and universal prognostic factors is an urgent need.

Comparison of survivors and non-survivors in the NHAP group showed difference in PC activity (Fig 2). The AUC of PC activity was the second highest in the comparison between PC activity and CURB-65 items. This means that PC activity has potential as a prognostic predictor for NHAP. Moreover, BUN lost its ability to predict mortality in NHAP when it was converted to a nominal variable at 20 mg/dL. This may also be related to NHAP patients being elderly and having many complications, and the cut-off values of existing scoring systems for CAP are insufficient for NHAP. In our study, the optimal cut-off of BUN for NHAP mortality was 27.4 mg/dL, with 66.7% of sensitivity and 77.8% of specificity. If the optimal cut-offs from this study were applied to each factor, the predictive power would increase further, but since the PSI and CURB-65 are widely used indexes, their cut-offs were left unchanged.

PSI and CURB-65 represent the total scores of various factors. Because direct comparison with PC activity alone is difficult, we compared the ROC curves of PC activity with those of each continuous variable included in CURB-65. PC activity was equivalent to BUN as the best of these factors (Fig 3). Because PC activity was a prognostic factor in NHAP (Table 4), we examined whether the utility of the PSI or CURB-65 for predicting the prognosis of NHAP could be improved by combining them with PC activity. In this analysis, we selected CURB-65 items as the existing and well-used scoring system for community acquired pneumonia; this combines CAP with NHAP, and it is called “all-pneumonia” in this study. In combining PC activity with the PSI, PSI is a comprehensive scoring system, and value such as 20 points was added for serum BUN ≥10.7 mmol/L, for example, therefore, we examined the optimal formula of PC activity with the ROC curve, and less than 55% was found to be applicable. The prognostic value of the scoring would be increased further if more optimal points were allocated; for example, by setting the cutoff for BUN at 27.5 mg/dL and using more than 20 points when assigned. However, since the PSI and CURB-65 are already widely used as prognostic scoring systems for pneumonia, and we did not change the cutoff or the assigned points; instead, we added the effect of PC activity. As a result, reclassification was found to be best with the addition of 25 points for PC activity <55% in the present study (S1 Fig). A score of 25 points was high compared to other components of PSI score, so we reclassified patients to modified PSI classes of five or six for scores ≥130. In addition, since PC activity turned out to be the useful predictor for prognosis of NHAP among CURB-65 items and PC activity, we examined the usefulness of making a full score of six for a modified CURB-65, comprising PC activity <55% for one point and the original factors of CURB-65. These modified PSI and modified CURB-65 appeared more useful than the original version for determining prognosis in NHAP cases, showing AUCs >0.730.

Regarding CAP, no difference in PC activity was found between survivors and non-survivors. This was considered to be due to the small number of deaths in this population. CAP patients were younger and had better activities of daily living than NHAP patients, so only 7.3% died in the present study. If the study had been designed to allow the recruitment of more CAP patients, PC activity may have been proven to be significantly effective in CAP cases as well. However, since PSI and CURB-65 have already been identified as useful for predicting CAP prognosis [14,15], the impact of PC activity on CAP would be smaller than on NHAP.

Several limitations of this study should be acknowledged. First, this investigation was performed in a single center. The background characteristics of NHAP patients can differ between facilities. Multi-center research would thus be desirable to clarify suitable PC activity threshold values. A multi-center study to validate the modified scoring systems is also warranted. Second, this study included hospitalized patients, but not outpatients. This study found that PC activity is useful for predicting the prognosis of patients hospitalized with NHAP. However, identifying outpatients who will develop a more serious status is considered one of the key objectives of this kind of biomarker research, so validation in more varied populations is needed. Measurement of PC activity is not yet widespread, but establishment of its utility would change this situation. Fourth, the effect of do-not resuscitate orders on mortality was not examined. Many patients who stay in nursing homes express do-not-resuscitate orders[46,47], and it is an important prognostic factor of pneumonia. However, given that none of the patients with NHAP received ventilatory management by intubation, our results would not have changed. Fifth, in the present study, the CURB-65 items were used as variables in the multivariate analysis because they have already been established as prognostic factors in community-acquired pneumonia in the broad sense of CAP and NHAP, which are grouped together as “all-pneumonia” in this study. However, there may be unknown confounding factors, and the discovery of useful prognostic factors for NHAP is needed in the future.

In conclusion, we showed that PC activity is a useful predictor of prognosis for NHAP. The PSI and CURB-65 were satisfactory for predicting the prognosis of CAP and all-pneumonia; when combined with PC activity, they provided good utility for NHAP. Further research is needed to determine the best formula to incorporate PC activity into prognostic systems for pneumonia, especially NHAP.

## Supporting information

S1 Fig The ROC curves of the PSI scores with additional scores for PC activity < 55% for predicting in-hospital death in NHAP. AUCs were compared by adding 20, 25, and 30 points to the PSI score when PC activity was less than 55%. The results showed the highest AUC when 25 points were added.
(TIF)

S1 File (XLSX)
