# Supplementary figures and images for: Protein C activity as a potential prognostic factor for nursing home-acquired pneumonia
Source: PLoS One. 2022 Oct 12;17(10):e0274685. doi: 10.1371/journal.pone.0274685 (PMC9555634; doi:10.1371/journal.pone.0274685)

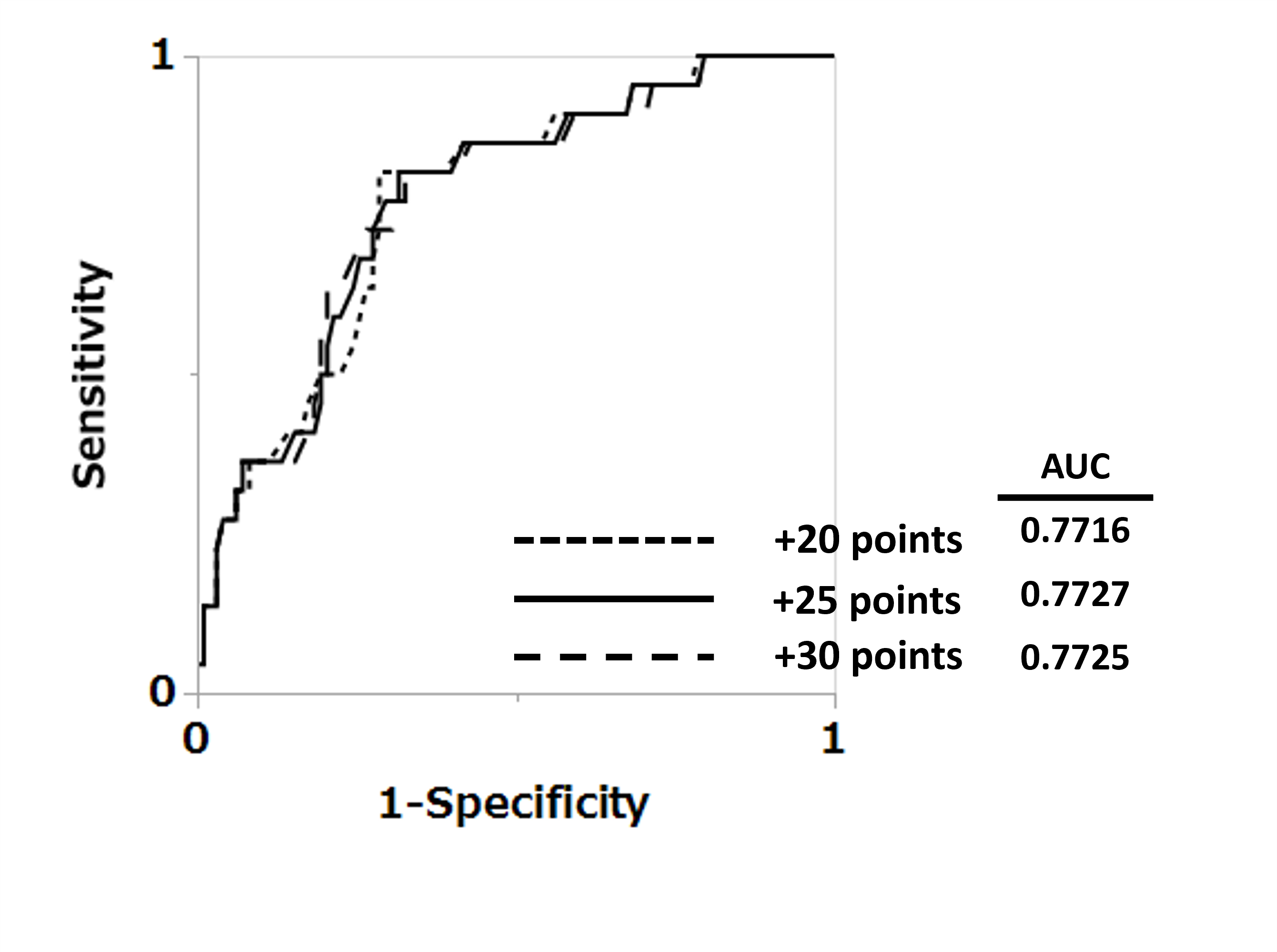

Supplement: S1 Fig — AUCs were compared by adding 20, 25, and 30 points to the PSI score when PC activity was less than 55%. The results showed the highest AUC when 25 points were added. (TIF) [file pone.0274685.s001.tif]
